# Rupture of 40-year-old silicone gel breast implants: a case report

**DOI:** 10.1186/s12877-023-04293-3

**Published:** 2023-09-23

**Authors:** Hsin-Hsuan Wu, Yu-Tse Weng, Yu-Yu Chou, Chih-Hsin Wang

**Affiliations:** 1 Department of General Medicine, Tri-Service General Hospital, National Defense Medical Center, Taipei, 114 Taiwan; 2 Division of Plastic and Reconstructive Surgery, Department of Surgery, Tri-Service General Hospital, National Defense Medical Center, Taipei, 114 Taiwan

**Keywords:** Breast augmentation, Breast implant rupture, Breast implant, Rupture, Silicone gel implant, Silicone gel

## Abstract

**Background:**

Implant rupture is one of the complications of breast augmentation surgery. The rupture of silicone implants is often insidious, potentially causing problems at any time. This is a case report of the rupture of 145-cc breast implants manufactured by Dow Corning Corporation and their removal at 40 years after augmentation.

**Case Presentation:**

A 70-year-old female patient was admitted for the removal of a lump in the upper and inner quadrants of the right breast. After a detailed examination, a rupture of the bilateral breast implants was diagnosed. Explantation without replacement was performed; the entire procedure proceeded smoothly. Immunohistochemical staining revealed siliconoma with lymphoid hyperplasia and calcification in the bilateral breasts with no signs of malignancy.

**Conclusions:**

Silicone breast augmentation is one of the most popular aesthetic surgical procedures worldwide. Therefore, it is important to educate patients on the need for close monitoring of their implants after augmentation through magnetic resonance imaging or ultrasound to facilitate early detection of any changes before a rupture occurs. Early detection of the implant rupture, in turn, will facilitate early and effective management.

## Background

Since its invention, silicone breast implants have become essential for aesthetic and reconstructive plastic surgery globally. According to the American Society of Plastic Surgeons, more than 300,000 breast augmentation procedures were performed in 2021, and breast augmentation has been among the top five cosmetic surgical procedures since 2006. In 2020, silicone and saline implants were used in 84% and 16% of all breast augmentations, respectively [1].

Implant rupture is a well-known complication of breast augmentation surgeries; breast implants cannot last forever. A previous study showed that an average rupture rate for silicone implants is 3–20 years [2]. The 10-year rupture-free implant survival rate for intact implants at 3 years is estimated to be 83–85%, with older implants expected to have poorer survival rates [2–4]. Core clinical trial data also divided the 10-year rupture rate according to different brands as follows: Allergan (Dublin, Ireland), 9.3–17.7%; Mentor (Irvine, CA, USA), 6.6–24.2%; and Sientra (Irvine, CA, USA), 8.7% [3]. Some risk factors for rupture include wearing underwire bras and seat belts and undergoing mammography [5]. Free silicone reacts with the surrounding tissues to form a fibrous capsule, owing to a natural foreign-body reaction [6]. The cohesiveness of the silicone gel allows it to stay within the capsular scar when the shell ruptures, thereby maintaining breast volume. This manifests as a ‘silent’ implant rupture as no clinically significant signs or symptoms are observed, which explains the unawareness among patients. Silicone implant ruptures were recorded in 8% of asymptomatic women [7] and 33% of symptomatic women [8]. Symptomatic patients often present with size change, pain, lump, breast capsular contracture, or asymmetry [3]. As a result, the U.S. Food and Drug Administration (FDA) has recommended magnetic resonance imaging (MRI) or ultrasound of silicone gel implants at 5–6 years after implantation, and every 2–3 years thereafter to screen for silent rupture [9]. Early detection will allow doctors and specialists to carry out early management of this condition. This case report discusses the rupture of breast implants and their removal 40 years after augmentation without regular follow-ups.

## Case presentation

**Fig. 1:**
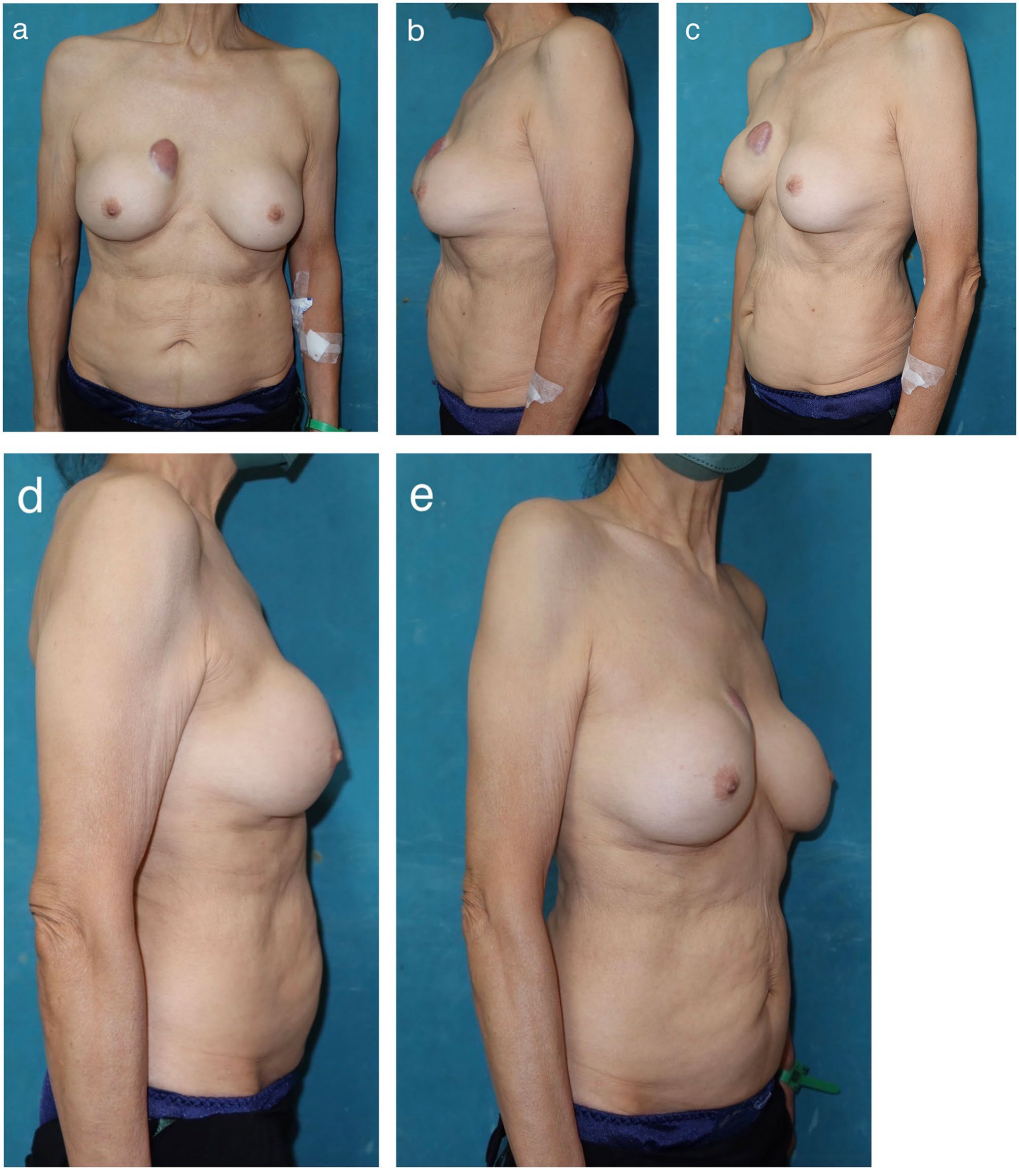
A 70-year-old female patient was admitted for implant explantation. (**a**) A painless mass associated with an erythematous skin change of approximately 3 cm in the upper and inner quadrants of the right breast that had persisted for years; progressive enlargement is noted. (**b**) Profile view of the left breast. (**c**) Quarter view of the left breast. (**d**) Profile view of the right breast. (**e**) Quarter view of the right breast

**Fig. 2:**
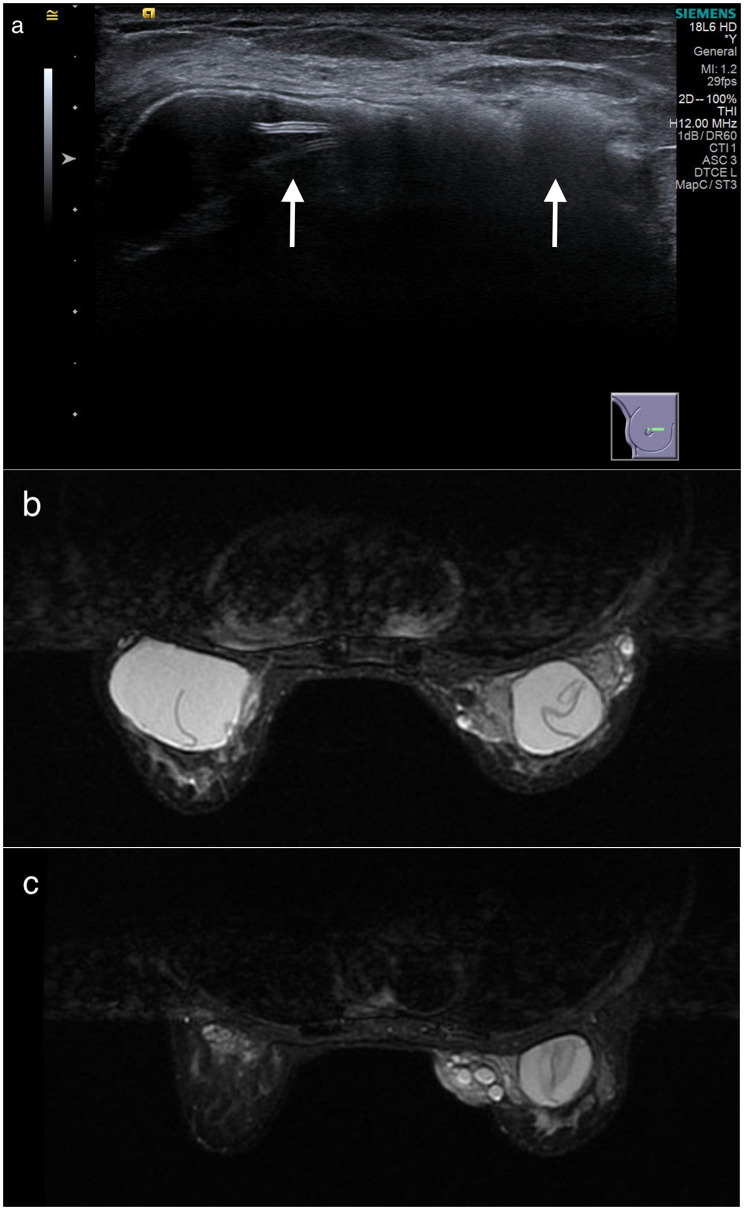
Diagnosis based on breast sonography and MRI. (**a**) Breast sonography shows intracapsular rupture of the implant. We observed a loss of the integrity of the shell with its fragments inside the implant (stepladder sign, left arrow). We observed an extracapsular rupture of the implant. The evident snowstorm pattern (right arrow) of the silicone outside the shell is characteristic of silicone outside the implant. (**b**) In MRI findings, the ‘linguine sign’ represents intracapsular rupture. (**c**) MRI shows that the ruptured implant migrated freely beyond the envelope and the fibrous capsule into the surrounding breast tissue. MRI, magnetic resonance imaging

A 70-year-old female patient presented with a lump in the upper and inner quadrants (UIQ) of the right breast. It was a painless mass associated with an erythematous skin change of approximately 3 cm and progressive enlargement (Fig. 1). She had undergone silicone gel-filled augmented mammoplasty at a private hospital in Taipei 40 years ago without any complications. After the augmentation, she did not undergo regular follow-ups for her breast implants. She had no medical or family history of breast cancer that would significantly affect her implants and no injury involving the chest area, and she had not received a breast massage. Physical examination revealed bilateral hardened breasts and palpable and visible implants. These signs and symptoms were consistent with capsular contractures corresponding to grade IV on the Baker scale. Breast sonography (Fig. 2a) revealed scattered hypoechoic nodules with sizes of < 5 mm in both breasts. Owing to the breast implants, fine-needle aspiration cytology was not attempted. Breast MRI (Fig. 2b, c) findings revealed silicone leakage around the right implant with focal fibrosis and a bulging configuration in the UIQ of the right breast. Concerned regarding the possible side effects of the silicone, the patient requested the implant to be removed without any replacement.

**Fig. 3:**
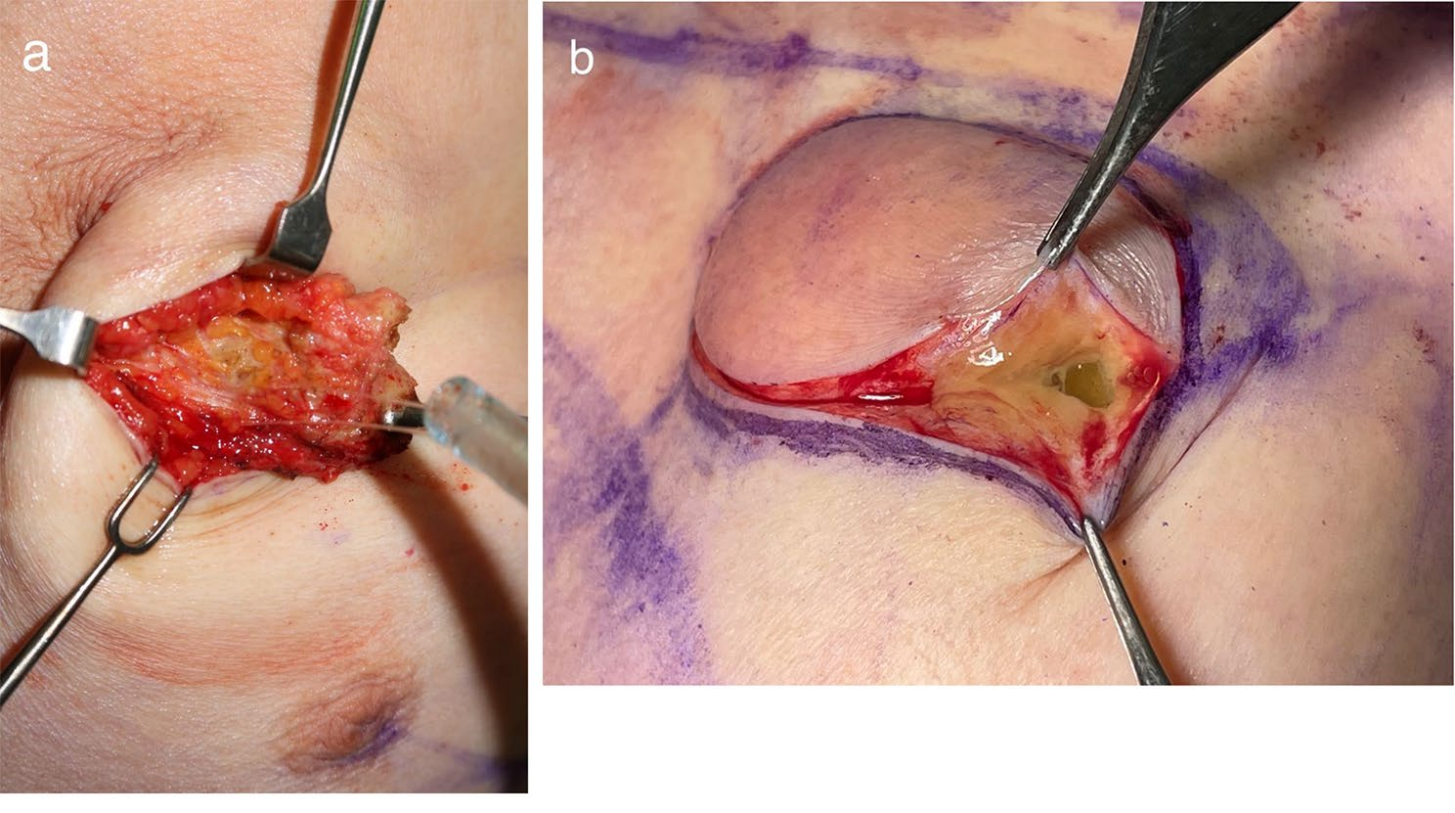
Operation findings. (**a**) A sticky gel-like material could be observed diffusing into the surrounding tissues of the left breast. (**b**) A gel-like material could be observed when dissecting through the upper and inner quadrants lump of the right breast

After obtaining informed consent, the breast tumour in her right breast was excised (Fig. 3b), both breast implants were removed, and capsulectomy was performed for both breasts (excision of the siliconoma of both breasts) in October 2022. The surgery was performed via her previous 3-cm inframammary incision. The implants and their surrounding capsules were removed from the subglandular layer. Next, the silicone gel was spread to the surrounding tissue (Fig. 3). Both implants were clinically intact, and the capsules were sent for pathological analysis.

**Fig. 4:**
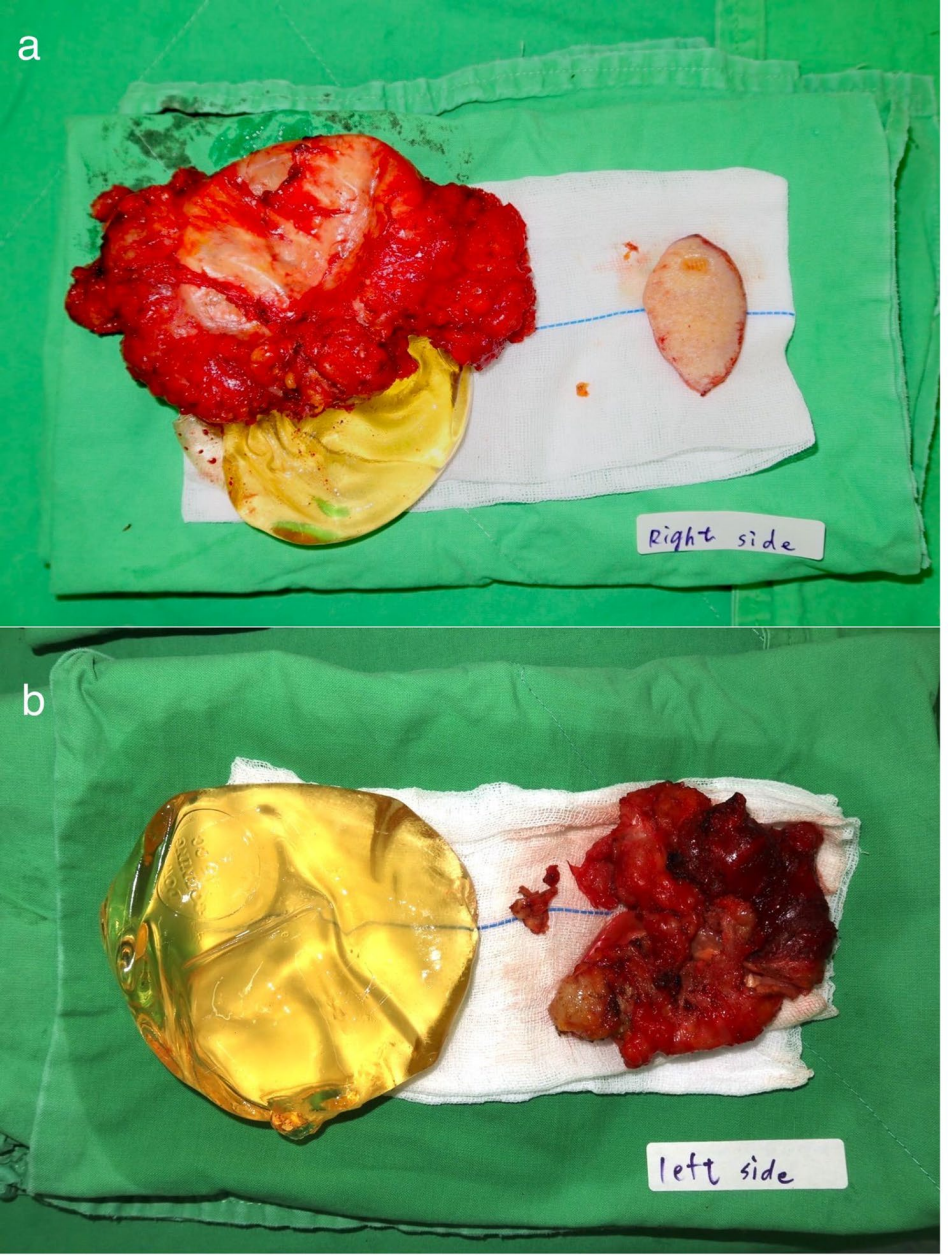
Removal of implants and tissues during surgery. (**a**) Implants and surrounding tissues formed a capsule, right. (**b**) Ruptured implant and capsule-forming tissue, left

Upon removal, the implants were reported to be 145-cc bilateral breast implants manufactured by Dow Corning Corporation (Fig. 4). The silicone gel was clear and yellowish. The shells were thick, without Dacron fixation patches on the posterior surface. Implants with this configuration were manufactured in the 1980s.

**Fig. 5:**
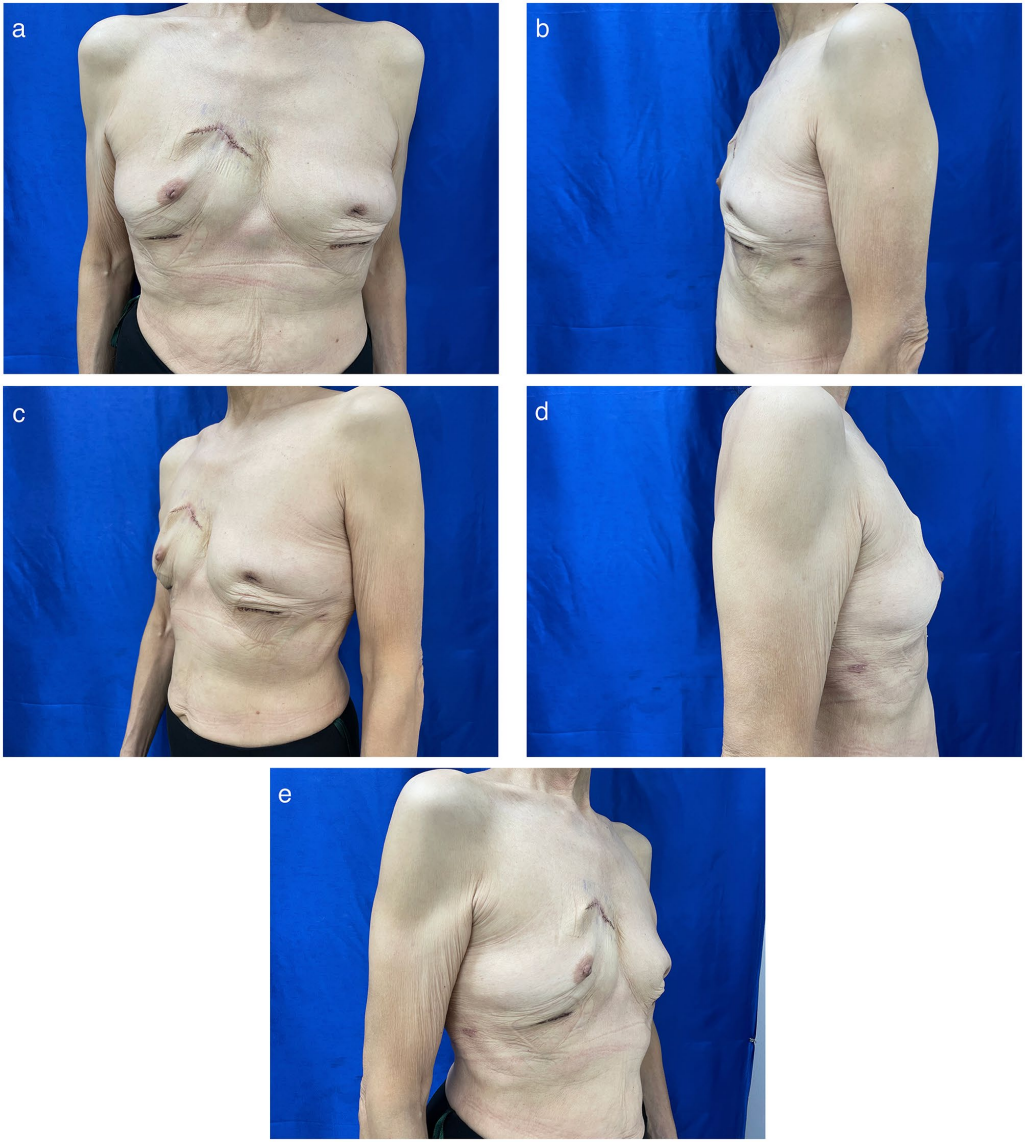
The status of the 70-year-old female post-implant explantation during follow-up at the outpatient clinic. (**a**) Front view. (**b**) Profile view of the left breast. (**c**) Quarter view of the left breast. **(d)** Profile view of the right breast. (**e**) Quarter view of the right breast

The patient had an uneventful postoperative recovery and was discharged the day after surgery. After 1 week, she visited our outpatient department for a follow-up examination. She did not exhibit or report any concerns regarding wound recovery. Immunohistochemical staining revealed siliconoma with lymphoid hyperplasia and calcification in the bilateral breasts with no signs of malignancy. However, the patient was satisfied with the postoperative results (Fig. 5).

## Discussion and conclusions

### Implant evolution

In the early 1960s, under the aegis of the Dow Corning Corporation, two surgeons, Frank Gerow and Thomas Cronin from Houston (Texas, USA) [10], proposed the first silicone implant. Over time, silicone gel-filled devices have undergone considerable advancements in design and have been subject to significant international scrutiny and controversy regarding their safety. Safety concerns ultimately resulted in a moratorium on silicone implants in the US from 1992 to 2000. Fourteen years later, silicone implants from two manufacturers, Mentor and Allergan, were approved by the FDA in November 2006 after research on long-term efficacy, safety, risk rates of rupture, capsular contracture, and reoperation; Sientra was later approved for augmentation and reconstruction [11].

The first generation of silicone implants from the 1960s included a thick silicone elastomer and thick silicone gel. Several Dacron fixation patches were fixed on the rear side to maintain the correct implant position. They had a firm consistency and were associated with a high incidence of capsular contracture and an increased risk of rupture due to the patches [12]. Consequently, second-generation implants were introduced in the 1970s to reduce the incidence of capsular contraction by incorporating thinner shells, less viscous gel fills, and Dacron-free patches; however, these increased the silicone bleed-through and rupture rates. In the early 1980s, third-generation implants focused on improving shell strength and permeability to reduce silicone bleed-through, implant rupture, and subsequent gel migration [13]. A fourth-generation implant, developed in the late 1980s, consisted of thick elastomer shells, more cohesive gel fills, and shell texturing. In the early 1990s, the fifth-generation implant devices pioneered an anatomical shape with a highly cohesive shape-stable gel filler and rough outer shell surface [14]. The textured surface aimed to improve implant stability and to prevent capsular contraction [15].

In our case, the patient had undergone breast augmentation 40 years ago, probably in the 1980s. Given the high rupture rate of second-generation implants, the implants used by our patient most likely belonged to the more durable third-generation implants produced by the Dow Corning Corporation.

### Diagnosis of implant rupture

One study showed that rupture of silicone implants occurred 12 years after implantation on average [16]. The degree of silicone implant rupture can be classified into 3 types: intracapsular, extracapsular or leakage. Intracapsular rupture refers to the silicone still confined within the fibrous capsule enveloping the implant. Extracapsular rupture takes place when visible silicone material is present outside the fibrous capsule that surrounds the implant. While leakage refers to silicone material permeating through the intact shell of the implant [17]. When a saline implant ruptures, the breast deflates, and salt water is absorbed by the body. In contrast, the detection of silicone implant rupture can be time consuming. The gel is not absorbed; thus, it spreads to the adjacent lymph nodes and lungs, forming granulomas in the arm, chest, or any other part of the body. Various diagnostic tools for implant rupture are available, including MRI, ultrasound, mammography, computed tomography, and physical examination. The latter is unreliable because it only has a sensitivity of 30% [6]. In contrast, MRI is ideal for detecting implant rupture, with a sensitivity of 100% and specificity of 54% for detecting rupture or leakage [18]. The ‘linguine sign’ has been reported to have a sensitivity of 93% and specificity of 65% in breakage detection. In comparison, ultrasound had a sensitivity of 67%, a specificity of 92%, and an accuracy of 77% in breakage detection [14]. FDA recommends MRI or ultrasound of silicone gel implants at 5–6 years after implantation and every 2–3 years to screen for silent rupture [9].

In our case, ultrasonography showed the stepladder and snowstorm signs (Fig. 2a), which indicated intracapsular and extracapsular rupture, respectively. In addition, the ‘linguine sign’ was present on breast MRI (Fig. 2b) findings, which revealed an intracapsular rupture of both breasts.

### Health implications

Health implications have been a concern for several studies. In most cases, an implant rupture does not appear to produce significant clinical symptoms or activate the humoral immune system. In rare cases, it can cause severe local problems [19]. However, these risks are inherent, as with any surgical procedure. Breast implants have been linked to lymphomas, autoimmune disorders, and systemic illnesses [20]. While ruptured silicone breast implants may cause silicone migration through the tissues, these foreign bodies can react with the surrounding tissues and form silicone granulomas, also known as siliconoma [21]. Siliconoma may occur locally or present at a distant site from the implant. It can create a firm mass, causing local tissue destruction, ulceration, scarring, or nerve damage [22]. Silicone lymphadenopathy is a complication resulting from distant migration to axillary or cervical lymph nodes. In pathological analysis, one of the lymph nodes showed histiocytic necrotising lymphadenitis (Kikuchi disease), which was interpreted as a localised immune response, owing to a sudden exposure to a large amount of silicones [23]. A previous study suggested the association of extracapsular silicone use with an increased risk of fibromyalgia. It is a disorder characterised by widespread pain, fatigue, and sleep disturbance [21].

There are insufficient studies for the health implications of silicone breast implant rupture. However, there are reports on distant silicone migration mimicking breast cancer, pneumonitis, pulmonary embolism, sarcoidosis, dermatomyositis, and hepatic congestion [17]. Nevertheless, owing to the rarity of these complications, the studies and cases on these diseases remain limited. Thus, it is important to inform patients about the potential long-term risk of breast augmentation.

In our case, the patient did not present for regular check-ups for her breast implants after undergoing augmentation 40 years ago. She became concerned when an erythematous lump appeared on the UIQ of her right breast. Thus, it is unclear to determine when the implant rupture exactly occurred. She eventually sought help after the lump started to increase in size. Fortunately, after removing the 40-year implant, the final pathological results showed a siliconoma with lymphoid hyperplasia in the bilateral breasts.

In conclusion, while silicone breast augmentation is one of the most popular aesthetic surgical procedures globally, they are not guaranteed to last a lifetime. Implant quality has improved significantly owing to advances in manufacturing, thereby reducing the risk of capsule formation and implant rupture. The rupture of silicone implants is often insidious, but it can lead to problems at any time. Therefore, it is important to educate patients on the need for close monitoring of their implants after augmentation through MRI or an ultrasound scan to facilitate early detection of any changes before a rupture occurs. Early detection of the implant rupture, in turn, will facilitate early and effective management.

## Data Availability

Not applicable.

## List of Abbreviations

FDA: Food and Drug Administration
MRI: Magnetic Resonance Imaging
UIQ: Upper and Inner Quadrants

## References

[1] Surgeons® ASoP. Plastic surgery statistics report. 2020. https://www.plasticsurgery.org/documents/News/Statistics/2020/plastic-surgery-statistics-full-report-2020.pdf. Accessed 20 November 2022.

[2] Hölmich LR, Friis S, Fryzek JP, Vejborg IM, Conrad C, Sletting S (2003). Incidence of silicone breast implant rupture. Arch Surg.

[3] Salzman MJ (2022). Silent rupture of silicone gel breast implants: high-resolution ultrasound scans and surveys of 584 women. Plast Reconstr Surg.

[4] Tanne JH (2006). FDA approves silicone breast implants 14 years after their withdrawal. BMJ.

[5] Paolini G, Firmani G, Briganti F, Macino M, Nigrelli S, Sorotos M (2023). Assessment of risk factors for rupture in breast reconstruction patients with macrotextured breast implants. Aesthetic Plast Surg.

[6] Hölmich LR, Fryzek JP, Kjøller K, Breiting VB, Jørgensen A, Krag C (2005). The diagnosis of silicone breast-implant rupture: clinical findings compared with findings at magnetic resonance imaging. Ann Plast Surg.

[7] Hedén P, Nava MB, van Tetering JP, Magalon G, Fourie le R, Brenner RJ (2006). Prevalence of rupture in inamed silicone breast implants. Plast Reconstr Surg.

[8] Chung KC, Malay S, Shauver MJ, Kim HM (2012). Economic analysis of screening strategies for rupture of silicone gel breast implants. Plast Reconstr Surg.

[9] Breast Implants - Certain Labeling Recommendations to Improve Patient Communication. Services USDoHaH, Administration FaD, Health CfDaR.; 2020. Contract No.: September 29, 2020.

[10] Glicenstein J (2005). Histoire de l’augmentation mammaire [History of augmentation mammaplasty]. Ann Chir Plast Esthet.

[11] Kessler DA (1992). The basis of the FDA’s decision on breast implants. N Engl J Med.

[12] di Pompeo FS, Paolini G, Firmani G, Sorotos M (2022). History of breast implants: back to the future. JPRAS Open.

[13] Maxwell GP, Gabriel A (2017). Breast implant design. Gland Surg.

[14] Gorczyca DP, Gorczyca SM, Gorczyca KL (2007). The diagnosis of silicone breast implant rupture. Plast Reconstr Surg.

[15] Retchkiman M, El-Khatib A, Nazhat Al Yafi M, Danino MA (2021). Biocell-initial patents versus user instructions guide: a discrepancy at the core of a crisis. Ann Chir Plast Esthet.

[16] Baek WY, Lew DH, Lee DW (2014). A retrospective analysis of ruptured breast implants. Arch Plast Surg.

[17] Paolo Montemurro, Pellegatta T, Burton H, Pafitanis G (2023). A case of ocular siliconoma and literature review. Aesthet Surg J.

[18] Maisel Lotan A, Retchkiman M, Tuchman I, Binenboym R, Gronovich Y (2016). Analysis of 109 consecutive explanted breast implants: correlation between suspected implant rupture and surgical findings. Aesthetic Plast Surg.

[19] Hölmich LR, Vejborg IM, Conrad C, Sletting S, Høier-Madsen M, Fryzek JP (2004). Untreated silicone breast implant rupture. Plast Reconstr Surg.

[20] Colwell AS, Mehrara B, Discussion (2019). Silicone implant illness: science versus myth?. Plast Reconstr Surg.

[21] Brown SL, Pennello G, Berg WA, Soo MS, Middleton MS (2001). Silicone gel breast implant rupture, extracapsular silicone, and health status in a population of women. J Rheumatol.

[22] Carson B, Cox S, Ismael H (2018). Giant siliconoma mimicking locally advanced breast cancer: a case report and review of literature. Int J Surg Case Rep.

[23] van Diest PJ, Beekman WH, Hage JJ (1998). Pathology of silicone leakage from breast implants. J Clin Pathol.

